# Alcohol, Hospital Discharge, and Socioeconomic Risk Factors for Default from Multidrug Resistant Tuberculosis Treatment in Rural South Africa: A Retrospective Cohort Study

**DOI:** 10.1371/journal.pone.0083480

**Published:** 2013-12-13

**Authors:** Emily A. Kendall, Danie Theron, Molly F. Franke, Paul van Helden, Thomas C. Victor, Megan B. Murray, Robin M. Warren, Karen R. Jacobson

**Affiliations:** 1 Division of Infectious Diseases, School of Medicine, Johns Hopkins Hospital, Baltimore, Maryland, United States of America; 2 Brewelskloof Hospital, Worcester, South Africa; 3 Department of Global Health and Social Medicine, Harvard Medical School, Boston, Massachusetts, United States of America; 4 Division of Molecular Biology and Human Genetics, Faculty of Medicine and Health Sciences, Stellenbosch University, Tygerberg, South Africa; 5 Department of Epidemiology, Harvard School of Public Health, Boston, Massachusetts, United States of America; 6 Section of Infectious Diseases, Boston University School of Medicine, Boston, Massachusetts, United States of America; McGill University, Canada

## Abstract

**Background:**

Default from multidrug-resistant tuberculosis (MDR-TB) treatment remains a major barrier to cure and epidemic control. We sought to identify patient risk factors for default from MDR-TB treatment and high-risk time periods for default in relation to hospitalization and transition to outpatient care.

**Methods:**

We retrospectively analyzed a cohort of 225 patients who initiated MDR-TB treatment between 2007 through 2010 at a rural TB hospital in the Western Cape Province, South Africa.

**Results:**

Fifty percent of patients were cured or completed treatment, 27% defaulted, 14% died, 4% failed treatment, and 5% transferred out. Recent alcohol use was common (63% of patients). In multivariable proportional hazards regression, older age (hazard ratio [HR]= 0.97 [95% confidence interval 0.94-0.99] per year of greater age), formal housing (HR=0.38 [0.19-0.78]), and steady employment (HR=0.41 [0.19-0.90]) were associated with decreased risk of default, while recent alcohol use (HR=2.1 [1.1-4.0]), recent drug use (HR=2.0 [1.0-3.6]), and Coloured (mixed ancestry) ethnicity (HR=2.3 [1.1-5.0]) were associated with increased risk of default (P<0.05). Defaults occurred throughout the first 18 months of the two-year treatment course but were especially frequent among alcohol users after discharge from the initial four-to-five-month in-hospital phase of treatment, with the highest default rates occurring among alcohol users within two months of discharge. Default rates during the first two months after discharge were also elevated for patients who received care from mobile clinics.

**Conclusions:**

Among patients who were not cured or did not complete MDR-TB treatment, the majority defaulted from treatment. Younger, economically-unstable patients and alcohol and drug users were particularly at risk. For alcohol users as well as mobile-clinic patients, the early outpatient treatment phase is a high-risk period for default that could be targeted in efforts to increase treatment completion rates.

## Introduction

Globally and in South Africa, the proportion of multidrug-resistant tuberculosis (MDR-TB) patients who successfully complete treatment remains less than 50% [1,2]. Completion of MDR-TB treatment is challenging due to its long duration (18-24 months), high pill burden, requirement of injections, and frequent side effects, leading many patients to default, defined as stopping regular treatment for two months or more prior to completing their treatment course. Default rates greater than 20% have been reported in South Africa [3,4]. The resulting incomplete treatment is associated with high patient mortality [5,6] and increasingly-resistant TB strains [7].

Identified risk factors for default from treatment of drug-sensitive (DS) TB include alcoholism and other substance abuse, low socioeconomic indicators, and health systems factors such as inadequate patient education and difficult-to-access care [8–10]. MDR-TB differs from DS-TB, however, with regard to both the prior treatment experience of the patients [11] and the increased difficulty and longer duration of the treatment regimen. Risk factors for default among patients treated for MDR-TB, particularly socioeconomic and substance-abuse risk factors and risk factors in rural communities, are incompletely characterized. 

Because of the required injections and the high pill burden, MDR-TB treatment is commonly initiated in the inpatient setting. Once patients are stabilized and have sputum that is smear-negative for *Mycobacterium tuberculosis*, and often after injectable medications are completed, patients are transitioned to the outpatient setting to complete more than a year of additional therapy. The inpatient and ambulatory settings create different sets of obstacles for patient adherence: ambulatory treatment disrupts patients’ lives less than hospitalization, but it requires their active participation and exposes them to competing pressures of family, livelihood, and substance use [12]. Additionally, the transition from inpatient to outpatient treatment may be a time of increased risk of loss to follow up, as has been observed for DS-TB in Moldova [13]. In this study, we sought to identify baseline risk factors for default from MDR-TB treatment and determine when during the treatment course default is most likely to occur, with special attention to the transition period between inpatient and outpatient treatment settings. 

## Methods

### Ethics Statement

The study protocol was approved by the Health Research Ethics Committee, Stellenbosch University, the Institutional Review Board of Boston University Medical Campus, and the Office of Human Research Administration, Harvard School of Public Health (exemption granted of additional review). The approving IRBs specifically waived the requirement of patient consent for information from their clinical records to be placed into a de-identified research database.

### Study Population

We conducted a retrospective cohort study of all adult patients (age > 15 years) who initiated MDR-TB treatment at Brewelskloof Hospital (BKH), the TB referral hospital for the Cape Winelands East and Overberg districts, Western Cape Province, South Africa, between 1 January 2007 and 31 December 2010. BKH serves a largely rural, agricultural population of approximately 600,000 people, and TB is one of the top three causes of death in all the region’s subdistricts, with 6714 cases of TB in 2008 and 74 cases of MDR-TB in 2008-09 [14]. Patients diagnosed with MDR-TB at general hospitals, local clinics, or mobile clinics in this region were referred to BKH to initiate treatment. MDR-TB was defined as infection with disease caused by *M. tuberculosis* resistant to both isoniazid and rifampin, as determined by culture-based or molecular-based (Genotype MTBDR*plus* line probe assay) susceptibility testing. 

At BKH, standard care for MDR-TB patients included hospitalization for the first four to five months of a 24-month treatment course. While in hospital, patients without contraindication to aminoglycosides received kanamycin injections, in addition to oral therapy of pyrazinamide, ethionamide, ofloxacin, and either ethambutol or terizadone. Patients remained in hospital until their sputum was smear and culture negative for two consecutive months, typically for a minimum of four months, although some early discharges were arranged for personal or disciplinary reasons. Patients then continued oral treatment as outpatients, typically at the health clinics where they were diagnosed. Depending on the distance between patients' homes and clinic, patients either went to clinic daily for directly-observed therapy (DOT), received medications every one to two weeks at clinic with community workers visiting their homes between clinic visits for home-based DOT, or, if they were particularly far from a clinic, received medications monthly from a mobile clinic and were not routinely linked to a community health worker. Care at clinics was carried out by nurses and supervised by BKH doctors who visited monthly. 

### Data collection

Both hospital and outpatient clinic records for all MDR-TB patients were maintained in a central office at BKH. BKH doctors collected social and economic variables on admission as part of their standard patient-history template. We abstracted hospital and clinic records in a de-identified manner using a standardized form for the following baseline variables: patient age, gender, race, height, and weight; human immunodeficiency virus (HIV) status, CD4 count, and antiretroviral therapy (ART) use; comorbid psychiatric disease and diabetes mellitus; recent alcohol, tobacco, and illicit drug use; marital status; employment status and type of employment; location of residence (in town versus on a farm), type of housing, and presence of electricity, running water, and toilet in the home; prior TB history including outcomes of previous treatment; and patients' current MDR-TB status including sputum collection dates and results, site(s) of disease, the clinic making the MDR-TB diagnosis and referral, dates of hospital admission and discharge, treatment regimens and dates, and final outcome with associated date. Subsequent, post-baseline measurements of these variables were not recorded. Employment was classified as “steady” if the patient was employed in a year-round (as opposed to a seasonal or day-labor) position or was a full-time student. Housing was classified as formal (a building of durable materials at an official address) or informal (a shack or shanty in a backyard or informal settlement), as the terms are used in South African housing surveys [15]. Alcohol, drug, and tobacco use were positive if there was recent use as recorded by the physician at intake. 

Outcomes were classified according to standard definitions [16]: cure or treatment completion required completion of prescribed therapy with or without microbiological confirmation of cure, respectively; default occurred if a patient missed two consecutive months of therapy for any reason, and was dated from the first day of the treatment interruption; outcome was death if a patient died of any cause while receiving MDR-TB treatment; failure occurred if a patient had persistently-positive culture results late in treatment, or if treatment was stopped early due to poor response or second-line drug resistance; and final outcome was transfer if a patient moved to another reporting and recording unit and the outcome of treatment was unknown. 

### Statistical Methods

We compared baseline patient characteristics between outcome groups, grouping cure with treatment completion and grouping death with failure in order to focus on default (Table 1). Age, body mass index, CD4 count, years of schooling, and time from sputum collection to treatment initiation were analyzed as continuous variables, and other variables were categorical. 

**Table 1:** Baseline characteristics of MDR-TB patients initiating treatment at Brewelskloof Hospital 2007-2010, overall and stratified by outcome category.

**Variable**	**All outcomes**	**N^a^**	**Outcome category^b^**			**P^c^**
			**Cure or Treatment Completion(N=113^d^)**	**Default(N=61^d^)**	**Death or Failure(N=43^d^)**	
Age (years, mean ± SD)	37.5± 11.9	225	38.1 ± 12.2	34.7 ± 10.5	40.7 ± 12.5	0.03
Female (N (%))	98 (44%)	225	54 (48%)	25 (41%)	17 (40%)	0.54
Coloured (N (%))	174 (77%)	225	87 (77%)	52 (85%)	31 (72%)	0.24
Married (N (%))	56 (26%)	219	37 (34%)	9 (15%)	8 (20%)	0.02
Formal dwelling (N (%))	154 (77%)	199	90 (88%)	35 (69%)	27 (69%)	0.004
Water, toilet, and electricity in dwelling (N (%))	116 (73%)	160	68 (80%)	26 (67%)	21 (62%)	0.08
Town (non-farm) address (N (%))	154 (69%)	223	81 (72%)	35 (58%)	33 (77%)	0.09
Diagnosed at mobile clinic (N (%))	19 (8%)	224	10 (9%)	9 (15%)	1 (2%)	0.10
Education (years, median (Q1, Q3))	7 (4, 10)	203	8 (5, 10)	7 (4, 9)	7 (4, 9)	0.23
Employed (N (%))	135 (64%)	212	69 (66%)	37 (62%)	26 (63%)	0.83
Steady employment (non-seasonal job, or student) (N (%))	51 (24%)	212	32 (31%)	7 (12%)	11 (27%)	0.02
Recent alcohol use (N (%))	134 (63%)	214	57 (54%)	47 (78%)	27 (63%)	0.01
Recent tobacco use (N (%))	157 (73%)	215	72 (67%)	50 (85%)	32 (74%)	0.05
Recent illicit drug use (N (%))	26 (13%)	203	7 (7%)	14 (25%)	4 (10%)	0.005
Current is first known TB episode (N (%))	30 (13%)	225	15 (13%)	7 (11%)	4 (9%)	0.78
Prior default from TB treatment (N (%))	49 (22%)	225	21 (19%)	18 (30%)	8 (19%)	0.21
Initial sputum smear AFB positive (N (%))	87 (43%)	204	45 (42%)	23 (46%)	16 (39%)	0.80
Extrapulmonary TB disease (N (%))	25 (11%)	220	9 (8%)	2 (3%)	12 (29%)	<.001
BMI (m^2^/kg)	18.8 ± 3.8	184	19.2 ± 4.2	18.4 ± 3.0	18.0 ± 3.7	0.21
HIV positive (N (%))	70 (32%)	222	30 (27%)	12 (20%)	24 (56%)	<.001
*CD4 count*	*141 (57, 273)*	*66/70*	*219 (88, 296)*	*199 (98, 489)*	*69 (39, 116)*	0.01
*On ARVs at MDR-TB treatment initiation* (*N (% of HIV infected*))	*13 (19%)*	*70/70*	*8 (27%)*	*1 (8%)*	*3 (7%)*	0.25
Diabetes mellitus at baseline (N (%))	8 (4%)	223	6 (5%)	1 (2%)	0 (0%)	0.17
Psychiatric comorbidity (N (%))	7 (3%)	225	5 (4%)	1 (2%)	1 (2%)	0.57
Days from sputum collection to MDR treatment initiation	65 (42, 94)	224	68 (51, 92)	67 (41, 117)	55 (32, 78)	0.07

^a^ Total number of patients with available (non-missing) data for variable

^b^ Transferred patients are excluded from this analysis.

^c^ P value is calculated for three-way comparison between “cure or treatment completion”, “default”, and “death or failure” groups, using ANOVA, Kruskal-Wallis rank sum, or Pearson’s Chi-square tests as appropriate.

^d^ Some values were missing for some variables.

We then examined baseline risk factors for time to default in univariable and multivariable Cox proportional hazard regression models. Time was measured from the first day of MDR treatment. Defaults were modeled as events, and other final outcomes were censored when they occurred. We verified proportional hazards assumptions by confirming that interactions between each covariate and time on treatment were not statistically significant, and by visual inspection of graphs of log(-log(survival)) versus log(time) stratified by each categorical variable. To generate a multivariable proportional hazards regression model for time to default, we considered all variables that were associated with default at P < 0.2 in univariate analysis, and we retained variables that were associated with default at P < 0.1 when added to the multivariate model. To account for missing data, we performed regression analyses using fivefold multiply-imputed datasets generated by Markov chain Monte Carlo methods (SAS MI procedure; SAS Institute), then pooled effect estimates across datasets [17].

To examine how the default rate changed over time and how the relationship between default and risk factors varied during treatment, we calculated default rates for three time periods: during hospitalization, within two months of discharge (“the early outpatient period”), and from two months after discharge until termination of treatment (“the late outpatient period”). We calculated these default rates for all patients and also when stratified by variables which we hypothesized would negatively impact treatment adherence in the outpatient setting: baseline recent alcohol use, whether the MDR-TB diagnosis was made in a stationary or mobile clinic, and whether the patient’s primary address was in town (indicating proximity to a local health clinic) versus a farm address (indicating a longer distance from routine TB care). We used tests of homogeneity to examine whether the associations between these risk factors and default differed by different treatment time periods (i.e., during hospitalization, the early outpatient period, and the late outpatient period).

We performed all statistical analyses using SAS version 9.3. 

## Results

### MDR-TB patient characteristics

We identified 225 MDR-TB patients who started treatment for MDR-TB at BKH between 1 January 2007 and 31 December 2010. MDR-TB was confirmed by culture or molecular resistance testing in 223 patients; two additional patients received empiric treatment based on previous culture history. 

Thirty-two percent of patients had HIV co-infection (Table 1). Thirty-six percent were unemployed at time of MDR-TB treatment enrollment, 24% had steady employment, and 40% worked seasonally or as day-laborers. Sixty-three percent of patients reported recent alcohol use, and 13% reported recent use of illicit drugs, typically marijuana or mandrax (the sedative-hypnotic methaqualone, mixed with marijuana and smoked). Eighty-three percent of patients had previously been treated for DS or DR TB, and 22% were known to have previously defaulted from TB treatment. Seventy-seven percent of patients were of Coloured (mixed ancestry) ethnicity, 22% were Black African, and two patients were White. Black patients were more likely than Coloured patients to be HIV infected (67% versus 22%) and live in informal housing (44% versus 17%); they were less likely than Coloured patients to use alcohol (41% versus 68%) or tobacco (44% versus 81%) (all p<0.01).

### Treatment outcomes and associated characteristics

Half of patients were either cured (86 patients, 38%) or completed treatment (27 patients, 12%). Sixty-one patients (27%) defaulted, 32 (14%) died during treatment, 11 (5%) failed treatment, and 8 (4%) transferred out. Comparisons of baseline characteristics between outcome groups are shown in Table 1.

With monthly sputum monitoring, 203 patients converted to negative sputum culture on treatment, and the mean time to first culture-negative sputum was 60 ± 47 days. Among the patients who defaulted, the sputum of fifty-four patients (89%) tested negative by culture before default, four patients (7%) remained positive by culture throughout MDR treatment for a median of 5 months (range 1 to 10 months) prior to defaulting, and three patients (5%) defaulted early before a second sputum was collected. 

### Risk factors for default

Univariate risk factors for default included alcohol, tobacco, and illicit drug use, younger age, unmarried status, lack of steady employment, informal dwelling, rural address, and diagnosis of MDR-TB at a mobile clinic (Table 2). In multivariable analysis, recent alcohol use (hazard ratio (HR)=2.1 [95% CI 1.1-4.0], P=0.02), recent drug use (HR=2.0 [1.0-3.6], P=0.03), and Coloured ethnicity (HR=2.3 [1.1-5.0], P=0.04) were risk factors for default, while formal housing (HR=0.38 [0.19-0.78], P=0.01), steady employment (HR=0.41 [0.19-0.90], P=0.03), and older age (HR=0.97 [0.94-0.99] per year of greater age, P=0.01), were associated with a lower rate of default (Table 2). 

**Table 2:** Single- and multivariable proportional-hazard regression models of default from MDR-TB treatment.

**Baseline variable**	**Hazard ratio (95% CI) in univariate analysis**	**P**	**Hazard ratio (95% CI) in multivariable model**	**P**
Recent illicit drug use	2.78 (1.48, 5.22)	0.001	2.02 (1.04, 3.95)	0.04
Recent alcohol use	2.24 (1.22, 4.13)	0.01	2.11 (1.11, 4.02)	0.02
Steady employment (non-seasonal job, or student)	0.37 (0.17, 0.80)	0.01	0.41 (0.19, 0.90)	0.03
Married	0.42 (0.21, 0.84)	0.01		
Formal dwelling	0.48 (0.25, 0.91)	0.03	0.38 (0.19, 0.78)	0.01
Age, per year of older age	0.98 ( 0.96, 1.00)	0.05	0.97 (0.94, 0.99)	0.01
Diagnosed at mobile clinic	2.00 (0.99, 4.07)	0.05		
Recent tobacco use	2.02 (1.01, 4.05)	0.05		
Town (non-farm) address	0.61 (0.37, 1.02)	0.06		
Prior default from TB treatment	1.66 (0.95, 2.87)	0.07		
BMI (per m^2^/kg)	0.94 (0.87, 1.01)	0.11		
HIV infected	0.61 (0.32, 1.14)	0.12		
Coloured ethnicity	1.72 (0.85, 3.49)	0.13	2.24 (1.03, 4.87)	0.04
Water, toilet, and electricity in patient’s dwelling	0.63 (0.33, 1.20)	0.17		
Education (per year of schooling)	0.97 (0.90, 1.03)	0.30		
Diabetes mellitus	0.36 (0.05, 2.69)	0.31		
Female	0.82 (0.49, 1.36)	0.44		
Initial sputum smear AFB positive at time of MDR-TB diagnosis	1.15 (0.67, 2.01)	0.60		
Number of prior TB episodes (per prior episode)	0.94 (0.72, 1.22)	0.64		
Current MDR-TB episode is patient’s first known TB episode	0.89 (0.40, 1.95)	0.76		
Employed (any employment)	0.95 (0.55, 1.62)	0.84		

Drug, alcohol, and tobacco use were intercorrelated (all associated pairwise with P<0.001), but alcohol use and drug use were independently associated with default. Although we found no statistically significant interaction between any covariate and time on treatment, visual inspection of graphs of the log(-log(survival)) versus log(time) suggested possible violations of the proportional hazards assumption for alcohol use as well as for MDR-TB diagnosis at a mobile clinic. 

### Timing of default

**Figure 1:**
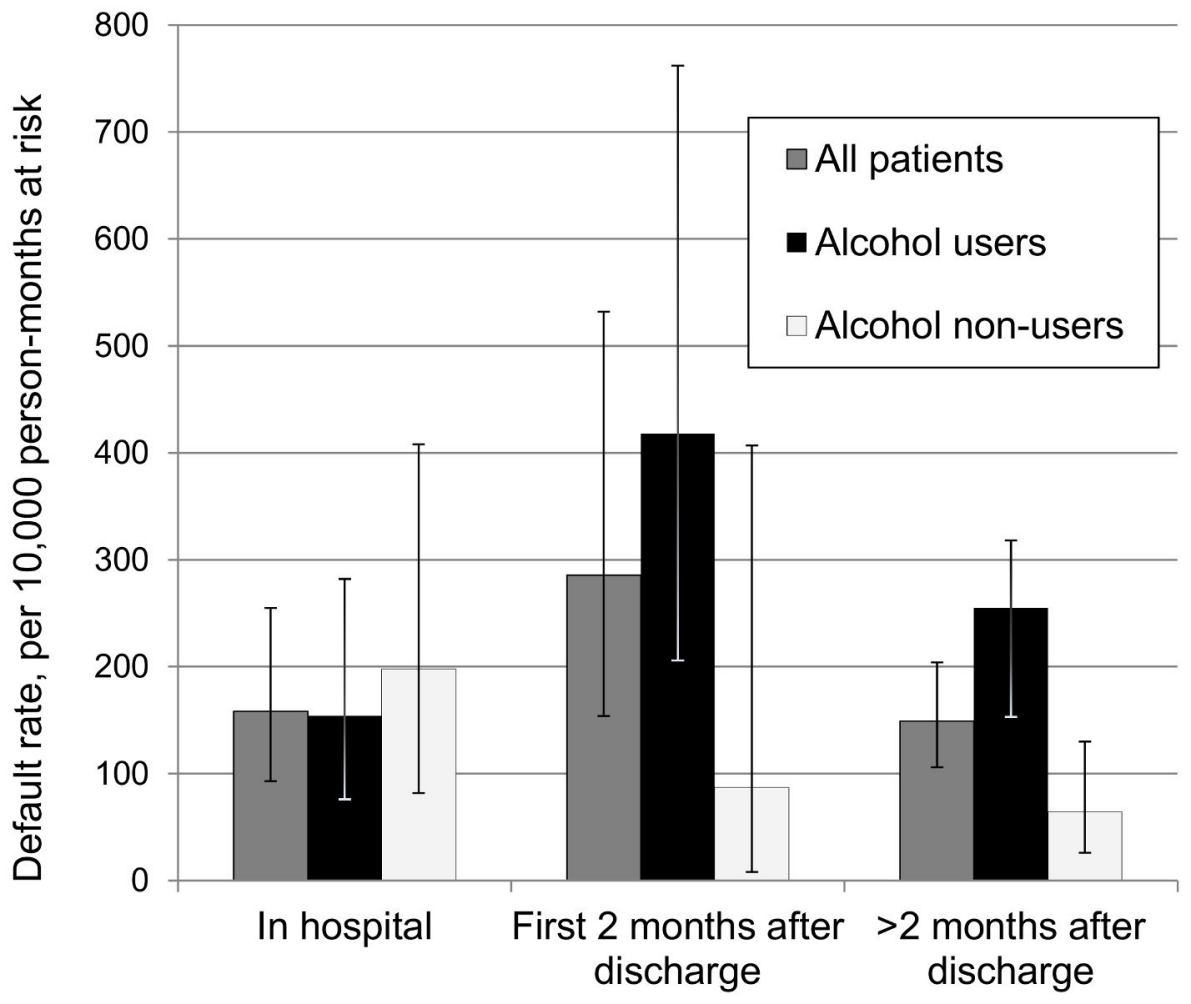
Default rates for patients on MDR-TB treatment, by time period and alcohol use status. Default rates with 95% confidence intervals were calculated in each of three time periods defined relative to hospital discharge date. Rates are shown for all patients as well as stratified by reported history of recent alcohol use.

Patients with outcomes of cure or treatment completion received median 739 (IQR 714-768) total days of treatment and were discharged from the hospital after median 127 (IQR 117-175) days. For patients who defaulted, median time to default was 257 days (interquartile range (IQR) 126-363 days). Of the 61 patients who defaulted, 15 (25%) defaulted during hospitalization–for example, by refusing further treatment or by failing to return from weekend leave. The default rate increased after discharge, and patients defaulted during the early outpatient period at 1.7 times the overall average default rate, although as discussed further below, no increased rate of default after discharge was seen in patients who had denied recent alcohol use (Figure 1). Only one patient defaulted after the eighteenth month of treatment. 

### Effect modifiers of post-discharge default risk

The relative risk of default associated with alcohol use varied with the hospitalization status (P=0.07 for test for homogeneity). During the in-hospital period, default rates were comparable among those who did and did not report recent alcohol use, but those with recent reported use at baseline defaulted nearly four times as frequently as nonusers during the early outpatient period and continued to default more frequently than nonusers during the late outpatient period (Figure 1). Among patients who did not report recent alcohol use, the default rate declined after the initial, in-hospital period. 

We also investigated whether patients with a farm address (versus a town address) or those who were diagnosed with MDR-TB at a mobile clinic (versus a hospital or local clinic) experienced higher rates of default after hospital discharge, since these patients may have more difficulty accessing care due to longer distances to health facilities and often do not receive daily DOT. In the early outpatient period, patients who had been diagnosed at mobile clinics defaulted at a seven times greater rate than other patients. This higher rate of default was confined to the early outpatient period (P=0.08 for test of homogeneity). We did not find any association between reporting recent alcohol use and being diagnosed at a mobile clinic (Pearson’s Chi-square P=0.58). Although having a farm address was associated with default in univariable analysis (Table 1), we did not detect effect modification by time relative to hospital discharge (P=0.41 for test of homogeneity). 

## Discussion

In our rural South African MDR-TB cohort, 27% of patients defaulted from treatment before completing a full 24-month course ‒ twice the 12-15% average default rate in published MDR-TB cohorts worldwide [18–20]. The high default rate we observed remains comparable to previously-observed South African MDR-TB treatment default rates of 29% in an urban Western Cape TB hospital in 1992-2002 [21] and of 21% in high-HIV-prevalence regions in 2000-2004 prior to antiretroviral availability [3,4]. Even if MDR-TB mortality and treatment failure decline with improved diagnostics, drugs, and other medical care, the 2015 target treatment success rate of 75% will remain unattainable unless these high rates of default also improve [1]. Recent South African MDR-TB guidelines recommend post-hoc interventions (psychological evaluations, substance abuse rehabilitation programs, or transportation or food aid) for patients who have demonstrated poor treatment compliance for corresponding reasons [22], but it would be preferable to allocate these resources effectively before defaults occur. Our findings demonstrate that certain socially-unstable patients are at risk for default and could benefit from adherence-focused interventions throughout their treatment. In addition, the association we found between alcohol use and post-discharge default highlights a patient population at particular risk as MDR-TB treatment delivery moves increasingly to the outpatient setting. 

Alcohol is known to be associated with developing active TB, as well as with poor clinical outcomes including drug resistance. Alcohol is also associated with default from treatment of DS TB, likely because of social destabilization caused by alcoholism, but possibly also in part because alcohol magnifies medication toxicities [23]. The few other MDR-TB cohorts to report substance use data have also noted substance use as a risk factor for treatment default: alcohol abuse was a risk factor in Eastern Europe [24,25], and substance (drug and/or alcohol) use was a risk factor in Peru and South Africa [6,26]. Our findings go further in demonstrating that a history of alcohol use may be especially predictive of default during the outpatient portion of treatment. We also show alcohol and drug use to be independent risk factors even after controlling for available socioeconomic variables. Alcohol use is prevalent in the Western Cape [27], and hospital-enforced abstinence may enhance initial treatment retention among alcohol users. Patients who use alcohol should be followed closely during the transition from hospital to outpatient treatment to ensure that they establish care with local clinics. Although financial supports such as free food and transportation reimbursement alone may not increase adherence in alcoholics [25], integrating medical or behavioral treatment of alcoholism into TB care has been shown to improve treatment adherence for some alcohol-using patients [28]. Drug use, although less common than alcohol use in our study population, may increase default by similar mechanisms and may be amenable to similar interventions. 

The association we found between informal housing and default agrees with previous identification of substandard housing conditions in Peru [6] and unstable residence in a five-province South African sample [26] as risk factors for MDR-TB treatment default – although the Peruvian study, which provided extensive social, financial, and psychiatric supports, had a much lower overall default rate of 10% [6]. In addition, we found that patients without steady employment, many of whom are seasonal farm workers in our study setting, are also at risk. To retain younger patients and patients with competing basic socioeconomic needs in care for the duration of MDR-TB treatment, targeted interventions to consider include community health worker support, DOT, and comprehensive financial support packages, all of which have demonstrated effectiveness [18]. 

We did not find associations between default and HIV or prior TB treatment. Although HIV was previously noted as a risk factor for default in South Africa prior to widespread antiretroviral treatment [3], HIV was associated with death but not default in our cohort, perhaps because many of these patients are also connected to the public health system for HIV as well as TB care. Prior default has been predictive of repeated default from DS-TB treatment [9,29], and although we saw some evidence of a similar trend in MDR-TB patients, the association was not significant in multivariable analysis and appears to be largely accounted for by other measurable risk factors such as substance use and employment status. 

The elevated default rates we observed in the two months immediately after discharge highlight the transition period between the inpatient and outpatient treatment settings as a high-risk time for MDR-TB treatment adherence. The increased rate of default in that period appears to be driven by individuals with a history of recent alcohol use. Also, although the sample size of patients using mobile clinics was small, having been diagnosed at a mobile clinic was also associated with a greater risk of default in the early outpatient period, and this association did not appear to be explained by alcohol use. South African guidelines recommend but do not require DOT beyond the intensive phase of therapy [22], and at the time of this study, most patients treated by mobile clinics did not receive daily DOT, which could facilitate the transition to outpatient care. Although high default rates following discharge have not been previously described for MDR-TB, a similar increase in default rates after discharge from the in-hospital, intensive treatment phase was reported among DS-TB patients in Moldova[13]. 

We note limitations to this study. Drug, tobacco, and alcohol use at intake were available for nearly all patients, but use was not quantified nor re-evaluated later during care. Quantification could help identify a threshold at which alcohol and drug use are problematic for adherence. It is known that drinking in this region tends to involve binging rather than moderate social use, and therefore, reported alcohol use often indicates abuse. In one survey of farm workers in the region, 87% screened positive for problem drinking by the CAGE questionnaire [27]. Women drink heavily as well; in a household survey, 68% of women who drank were classified as high-risk drinkers by the Alcohol Use Disorders Identification Test (AUDIT) scale [30]. Had we been able to distinguish between high-risk and more moderate alcohol use, we may have observed a stronger association between high-risk alcohol use and default than that which we observed for any recent alcohol use. 

We were unable to enroll or quantify those patients diagnosed with MDR-TB who never started treatment. Only 44% of the South African patients who were diagnosed with MDR-TB in 2011 initiated treatment [31], and the risk factors for never entering care may differ from the risk factors for starting but not completing treatment. We also cannot determine whether associations we observed were causal, although these associations are mechanistically plausible. Lastly, we had limited information about patients’ access to clinics; we used farm addresses and mobile clinic diagnoses as proxies for longer distances to clinic. Precise travel times to clinic and data about frequency of health worker contact would help clarify the extent to which proximity to health clinics affects default. 

Default is a major barrier to cure in MDR-TB patients, and in this rural farming population, younger patients, economically-unstable patients, patients receiving care from mobile clinics, and alcohol and drug users are particularly at risk for default. Socioeconomic supports targeting the most at-risk groups may help to increase treatment adherence in the Western Cape and similar areas of high MDR-TB burden. Also, although outpatient treatment may be easier, more affordable, and better for patients in the long run, the transition from in-hospital to community-based treatment is a difficult period in which extra care must be taken to prevent default. Because of alcohol-using patients’ higher default rate during the transition period and in the outpatient setting, MDR-TB programs should assess alcohol use patterns in their patient populations and consider following and supporting alcohol-using patients more intensively. 
